# Increased dose primary thromboprophylaxis in ambulatory patients with advanced pancreatic ductal adenocarcinoma, a single centre cohort study

**DOI:** 10.1186/s12959-020-00222-1

**Published:** 2020-05-29

**Authors:** Anthony Maraveyas, Farzana Haque, Iqtedar Ahmed Muazzam, Waqas Ilyas, George Bozas

**Affiliations:** 1 Queen’s Centre for Oncology and Haematology, Castle Hill Hospital, Hull University Teachng Hospitals (HUTH), Cottingham, HU16 5JQ UK; 2 Joint Centre for Cancer Studies, Faculty of Health Sciences, The Hull York Medical School, University of Hull, Cottingham, HU6 7RX UK

**Keywords:** Pancreatic cancer, Thromboprophylaxis, Dalteparin, Ambulatory, Chemotherapy

## Abstract

**Background:**

Advanced pancreatic ductal adenocarcinoma (aPDAC) patients have a lifetime all type thromboembolic event (ATTE) rate of 25–35%. Efficacy and safety of increased dose primary thromboprophylaxis (IDPTP) with low molecular heparin (LMWH) given for 3 months has been shown in two prospective randomized trials.

**Objectives:**

To report on efficacy -reduction of all type thromboembolic events (ATTE)-, safety -incidence of Major Bleeding (MB)- and compliance in a single-centre cohort of aPDAC patients receiving first line chemotherapy and LMWH-IDPTP.

**Methods:**

From May 2009 to October 2016, 82 patients received IDPTP –LMWH with dalteparin. Schedule: 55 kg and below: 7500 IU, between 55 and 80 kg: 10,000 IU, above 80 kg: 12,500 IU. MB is reported using the International Society of Thrombosis and Haemostasis (ISTH) criteria. ATTE was defined as any arterial or venous event, incidental or clinically symptomatic, including visceral VTE.

**Results:**

Mean and median time on dalteparin was 10.2 (95%CI 8.1, 12.4) and 8.0 (95%CI 6.2, 9.7) months respectively. ATTE was observed in 7 (8.5%) of patients, with a median time on IDPTP of 6.2 months (95% CI 10.0, 13.2). MB was seen in 10 (12.2%) patients with a median time on IDPTP of 4.5 months (95% CI 1.6, 7.4). Six major bleeds (60%) were the direct or indirect result of aPDAC. Eighty-one patients had died at the time of data collection with a median overall survival time of 8.7 months (95%CI 6.4, 11.0). Thromboembolism and bleeding were late events. No impact of thromboembolism or bleeding on overall survival was observed.

**Conclusions:**

IDPTP-dalteparin was associated with lower ATTE occurrence rates than expected and comparable major bleeding rates. ATTE and MB were late events, the majority of MB was from direct or indirect result of locally progressing aPDAC. Since these conditions can frequently arise in aPDAC, IDPTP should be regularly reviewed beyond 3 months.

## Introduction

Patients with advanced pancreatic ductal adenocarcinoma (aPDAC) have a seven-fold increased risk of developing arterial and venous thromboembolism as compared to patients with most other solid and haematological malignancies [1, 2]. This translates to about one-third of patients with aPDAC suffering from either a clinically evident or unsuspected (*incidental*) thrombo-embolic event (TE) over short life spans, and the spectrum of these events is broad, ranging from the conventional venous thromboembolism (VTE) of deep vein thrombosis (DVT)-pulmonary embolism (PE) complex, to arterial as well as visceral thromboses [3]. The manifestation of a VTE, apart from the physical and psychological burden inflicted on the cancer patient, generally results in increased health utilization costs of up to 45% per affected cancer patient [4] with VTE incremental costs being the highest in pancreatic cancer patients [5].

The administration of chemotherapy in ambulatory aPDAC patients is an additional risk factor for thromboembolism which further complicates an already challenging management [3]. A modest improvement in survival has been seen with new chemotherapy combinations [6] but they have also resulted in increased VTE risk [7]. The use of currently available anticoagulant agents reduces VTE risk and their efficacy has been proven in randomized trials [8, 9]. Current guidelines from major international bodies have changed recently to suggest that patients with Khorana Score 2 and above should be considered for thromboprophylaxis. This by definition includes patients with pancreatic cancer [10–12]. These guidelines have been updated following the studies AVERT [13, 14] and CASSINI [14, 13] and now include the option of using a direct oral anticoagulant (DOAC). The recent (2018) NICE guidance also suggests that thromboprophylaxis should be considered in aPDAC [13, 15]. Both the FRAGEM study [8] with a 19.6% absolute difference in thromboembolic rates and a number needed to treat (NNT) of 5 and CONKO-04 [9] with an absolute difference of 8.7% in thromboembolic rate and a NNT of 11 provide meaningful clinical benefit.

These studies used increased dose primary thromboprophylaxis (IDPTP) for 3 months. The investigators in CONKO 004 used a two-third -therapeutic dose of enoxaparin (1 mg/kg once daily) for the first 3 months, followed by conventional primary prophylaxis maintenance of 40 mg sc. once daily. The investigators in FRAGEM used full therapeutic dose schedule of dalteparin (200u/kg once daily for 4 weeks reduced to 150u/kg once daily) for the first 3 months at which point thromboprophylaxis was discontinued.

None of the current guidelines offer a clear opinion on LMWH dosing though the ASCO guideline mentions that the studies of LMWH thromboprophylaxis in pancreatic cancer patients were conducted at a ‘treatment dose’ of LMWH [10] otherwise termed ‘supra-prophylactic’ [16].

Soon after the LMWH–related data were first published Hull University Teaching Hospitals (HUTH) implemented a dalteparin-based thromboprophyalxis schedule for aPDAC patients using a supra-prophylactic dosing approach. It is this single-centre retrospective real-world cohort series experience in ambulatory aPDAC patients that we present here.

### Objectives

To report efficacy, safety and compliance in a real-world cohort of consecutively treated patients with advanced and metastatic pancreatic cancer receiving an increased dose primary thromboprophylaxis (IDPTP) schedule of dalteparin.

## Patients and methods

### Increased dose primary thromboprophylaxis (IDPTP) schedule

For aPDAC patients deemed fit for systemic treatment we implement a simplified dalteparin increased dose thromboprophylaxis schedule, close to the 75% full therapeutic dose of the CLOT schedule used in the FRAGEM trial and in accordance with the CONKO-04 scheduling of continuing thromboprophylaxis throughout chemotherapy but without mandating a switch to conventional dose or discontinuation.

The IDPTP dalteparin schedule was weight adjusted and ranged as follows. For patients 55 kg and below: 7500 IU, for patients between 55 and 80 kg: 10,000 IU and for patients above 80 kg: 12,500 IU.

### Patients

The HPB-MDT database (Taunton & Somerset NHSFT QGCE 1499) was searched for all pancreatic cancer patients that were referred to the cancer centre between May 2009 and October 2016, and then cross referenced with the electronic chemotherapy database (ARIA MedOnc 13.7). Patients were further cross referenced through the electronic medical record system (iSOFT Patient Centre®, CSC™ and Lorenzo®, CSC™) and through the pharmacy dispensary database (Ascribe RX™) for all the outpatient prescriptions for anticoagulants. From these electronic medical records demographic, historic, cancer therapy and cancer specific data were collected for each patient in a retrospective manner. All patients had histological/cytological proof of ductal adenocarcinoma of the pancreas with inoperable locally advanced or metastatic cancer and an multi-disciplinary team (MDT) decision for systemic treatment. Only patients due to receive first line treatment were included. Accordingly, patients had to have haematological and biochemical indices allowing systemic treatment for their cancer to be eligible for treatment with IDPTP. Non-ambulatory patients or patients not considered for systemic cancer treatment either due to performance status or non -alleviated cancer morbidity (e.g. non-relievable obstruction of biliary tree or duodenal outlet) were excluded. Patients with any contraindications for anticoagulation (dalteparin) were excluded. Patients receiving other anticoagulation (e.g. a direct oral anticoagulant or warfarin) and not transferred to IDPTP were excluded.

### Outcome measures

Primary aim of the study was to determine the occurrence rate of all type thromboembolic events (ATTE) and rate of major bleeding (MB). ATTE was defined as any TE event -arterial or venous, incidental or clinically symptomatic including visceral VTE and is reported in this manuscript as ATTE or TE. VTE is used for conventional PE/DVT definitions. All recorded ATTE and bleeding events were reviewed by three of the authors (AM, GB & IM) and the nature, severity and classification agreed. Bleeding episodes were classified into major and minor by ISTH criteria. Major bleeding was defined as any fatal bleeding, symptomatic bleeding in a critical area or organ, such as intracranial, intraspinal, intraocular, retroperitoneal, intra-articular or pericardial, or intramuscular with compartment syndrome, and/or bleeding causing a fall in haemoglobin level of 20 g L^− 1^ (1.24 mmol L^− 1^) or more, or leading to transfusion of two or more units of whole blood or red cells [17]. Compliance to anticoagulant treatment was inferred from the time and length of the last recorded outpatient or ward prescription chart.

### Approvals & Statistics

The study reported in this manuscript is the result of work that has been classified as an audit. This is a regular undertaking in UK hospitals with the primary goal of maintaining quality standards and identifying areas that need improvement if they fall below accepted standards as set by national or international guidelines. Audits can be retrospective or prospective. As per the NHS Health Research Authority guidelines our study, which can be classified within the audit / service evaluation description, does not require external Research Ethics Committee approval [18]. The NHS Trust governance body which authorises the project is doing so if the study is conducted within the regulatory framework including the Data Protection Act (1998), the Caldicott principles (1997) and the NHS Confidentiality code of practice (2003) [19]. Within this context regulatory approval is sought and obtained based on the quality of the audit and the priority of the area studied and whether it fits within the quality framework of the organization. The endorsement code for our study is 2017.98 issued by the Hull and East Yorkshire Hospitals NHS Trust approved the 1st June 2017.

Patients were registered sequentially on the database. Data were collected in an MS EXCEL®2010 (Microsoft Corp™) spread sheet maintained in a secure virtual hard-drive with restricted access. All analyses were performed with SPSS ver22, IBM Corp®. Descriptive statistics were used to analyse patient characteristics. Data on the TE-free interval (TFI) and bleeding-free interval (BFI) were reported in months from the time of commencing IDPTP up to the index event (last follow-up contact or the date of death). The collected data was also used to calculate the median overall survival of the whole study population and whether TE and bleeding episodes had demonstrated any impact on survival. No patients were lost to follow-up. To correct for competing risk of death the rate of TE or major bleeding for the time intervals was calculated as the number of events divided by the total number of patients at risk (subject months). The Clopper-Pearson method was used for 95% confidence interval. The Kaplan Meier method was utilised to explore the prognostic significance of categorical variables using the log rank test to compare factors.

## Results

**Fig. 1:**
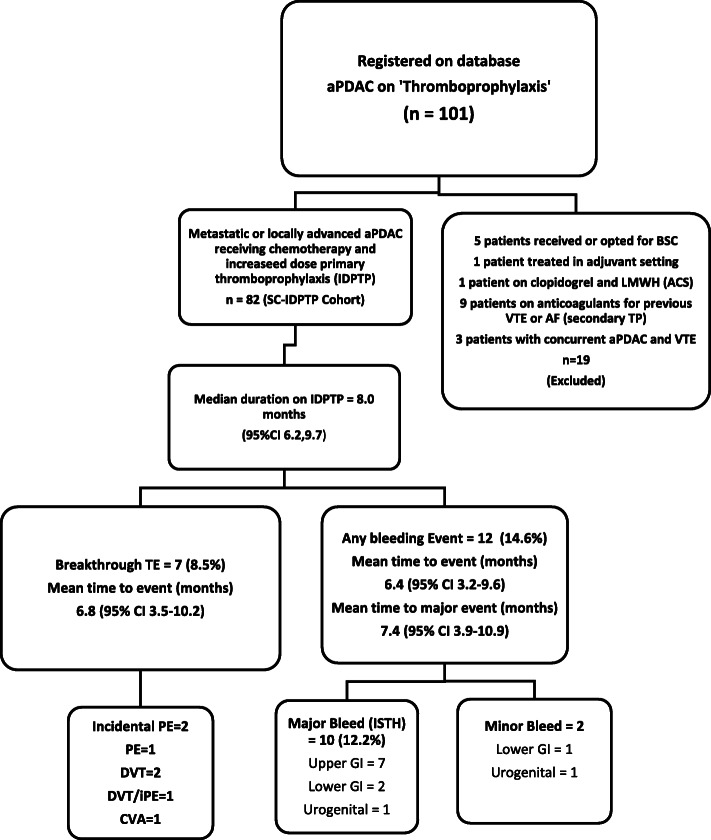
Study Flow Chart. Patients that were identified using the search strategy described in the methods were entered on to a data base. Their case notes were reviewed and the patients that bore similarities to the experimental cohort of patients treated in the FRAGEM [8] and CONKO-04 [9] studies were selected for further analyses (IDPTP group). The miscellaneous reasons for exclusion are detailed. (ACS = Acute Coronary Syndrome, AF = Atrial Fibrillation, aPDAC = advanced Pancreatic Ductal Adenocarcinoma, BSC = Best Supportive Care, CVA = Cerebrovascular Accident, DVT = Deep Vein Thrombosis, GI = Gastrointestinal, iPE = Incidental Pulmonary Embolism, LMWH = Low Molecular Weight Heparin, PE = Pulmonary Embolism, SC-IDPTP = Study Cohort of Increased Dose Primary Thromboprophylaxis, TE = Thromboembolic Event, TP = Thromboprophylaxis, VTE = Venous Thromboembolism)

One hundred and one patients with aPDAC were registered on the database. The search process also identified patients with prior VTE either before cancer diagnosis or during adjuvant treatment for PDAC, patients with concurrent diagnosis of TE and aPDAC or patients on long term anticoagulants due to pre-existing AF (atrial Fibrillation) or unprovoked VTE having been switched to an IDPTP schedule (details in Flow chart Fig. 1).

Demographics and event rates are reported for the whole cohort but the detailed analyses are restricted to the 82 patients that corresponded to an entirely IDPTP cohort.

The median age was 67 years, interquartile range: 61–72 (*n* = 82) and 54% were male. Sixty five per cent of these patients were metastatic and the rest locally advanced. Ninety-six patients were offered first line chemotherapy. Sixty nine per cent of the patients received only one line of treatment, 20% received two lines of treatment and only 2% received a third line of treatment.

Of the IDPTP cohort (*N* = 82) 64 patients (78%) were offered Gemcitabine based first line treatment (48 patients (58%) single agent gemcitabine & 16 (20%) first line gemcitabine-based combination) and 18 patients FOLFIRINOX.

The baseline characteristics of all patients, for the 82 IDPTP patients and the primary endpoint data are shown in Table 1.

**Table 1:** Patient Characteristics and Events (Major and Minor Bleeding & All-type Thromboembolic Events). Data shown for both the IDPTP Cohort and for thepatients that were excluded

	Miscellaneous TP	IDPTP
**Patients**	19	82
Age (years)	71 (r41)	67 (r51)
Male	11 (58%)	43 (52%)
Metastatic	10 (53%)	56 (68%)
WHO PS 0–1	10 (53%)	67 (82%)
**Chemotherapy**	^a^14 (73%)	^b^82 (100%)
FOLFIRINOX	2 (10%)	18 (22%)
Gemcitabine SA	10 (52%)	48 (58%)
Gem-Based Combi	1 (5%)	16 (20%)
No Chemo	5 (27%)	–
**Events (Bleeding)**	4 (20%)	12 (14.5%)
#Major Bleeding	2 (10%)	10 (12.2%)
Upper GI bleed	2 (100%)	7 (70%)
Lower GI bleed	–	2 (20%)
Genitourinary		1 (10%)
#Minor Bleeding	2 (10%)	^c^2 (2.5%)
**Events (AT-TE)**	7 (37%)	7 (8.5%)
DVT	2	2
i-PE	2	2
PE	–	1
i-PE & DVT	–	1
CVA	2	1
DVT & Visceral	1	

^a^One patient was treated with adjuvant chemotherapy (5FU/LV)

^b^73% completed one line, 24% two lines & 2.4% three lines of chemotherapy

^c^1 minor bleed was upper GI

**Fig. 2:**
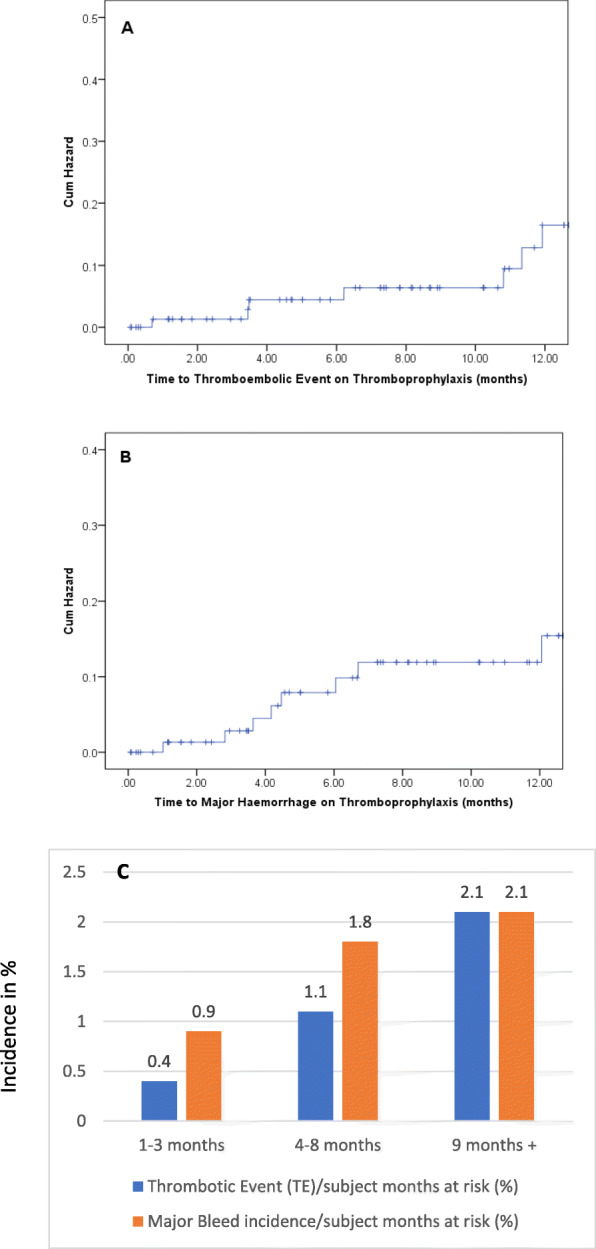
The Hazard Plots (Kaplan Meier) for: **a** TE events, (**b**) Major Haemorrhage for the first 12 months of follow-up of 82 patients on IDPTP. **c** Incidence of thrombembolic events (TE) and Major Bleeding at different time periods. A lower incidence of TE is seen during the 1st 3 months, 0.4% (95% CI 0.0, 2.4; 1/226 subject months), compared to 2.1% (95% CI 0.4, 5.9; 3/146 subject months) after 8 months (the median duration of thromboprophylaxis in this group). A similar pattern is notable for major bleeding. Incidence was less in the 1st 3 months, 0.9% (95% CI 0.1, 3.1; 2/227 subject months). During the 4–8 months period (280 subject months), 5 patients experienced a major bleeding event that gives an incidence of 1.8% (95% CI 0.6, 4.1). Comparable to that, 3 patients (146 subject months) had major bleed after 8 months with an incidence of 2.1% (95% CI 0.4, 5.9)

In the IDPTP cohort (*n* = 82), patients remained on thromboprophylaxis for a median duration of 8.0 months,95% CI (6.2, 9.7). Seven patients [8.5, 95%CI (3.7,14.6)] developed a TE despite IDPTP and ten patients [12.2, 95%CI (6.1,19.5)] in the IDPTP group had a major bleed and 2 patients [2.4 95%CI (.0,6.1)] had a minor bleed, for a combined event rate of major bleeding and TE of 18% [*n* = 15/82 as two patients had a TE and a bleed, 95%CI (9.8,26.80)]. The correlation between the IDPTP and the timing of TE and bleeding can be seen in Fig. 2. The cumulative rates of MB at 3 and 12 months of this real-world cohort are compared to the experimental arms of FRAGEM and CONKO-04 where data were available (Table 2). Figure 2a & b present the cumulative hazard of developing a breakthrough TE or bleed. The accelerated phase of the cumulative hazards plot is between 4 and 8 months which is later than the conventional first 3 months seen in patients without thromboprophylaxis, reflecting the median time to TE of 6.2 months (95% CI 10.0, 13.2). This can be seen most clearly in Fig. 2c where the data are corrected for competing risk of death. A similar late pattern is noted for the major bleeding episodes median time to MB 4.5 months (95% CI 1.6, 7.4). The majority of the minor and major bleeding events were upper GI (8/14, 57%) and were directly or indirectly attributable to an aPDAC related cause.

**Table 2:** Cross-study comparison of three- and 12 month landmark events (Major Bleeding and Thromboembolism)

EVENTS (Cumulative Incidence)	CONKO-04	FRAGEM	SC-IDPTP
**Major Bleed (3 Months)**	4.5%(7/160)	3%(3/59)	2.4% (2/82)
**Major Bleed (12 months)**	8.3%(13/160)	Not Reported (N/R)	9.7% (8/82)
**Breakthrough PE/DVT (12 months)**	5.7%(9/160)	^a^8.4% (5/59)	^b^4.8% (4/82)
**Breakthrough All type TE (12 months)**	Not Reported (N/R)	12% (7/59)	8.5% (7/82)

^a^Excludes 2 cases of I-PE to be comparable to CONKO-04

^b^Excludes 2 cases of I-PE and the CVA so as to be comparable to CONKO-04

**Fig. 3:**
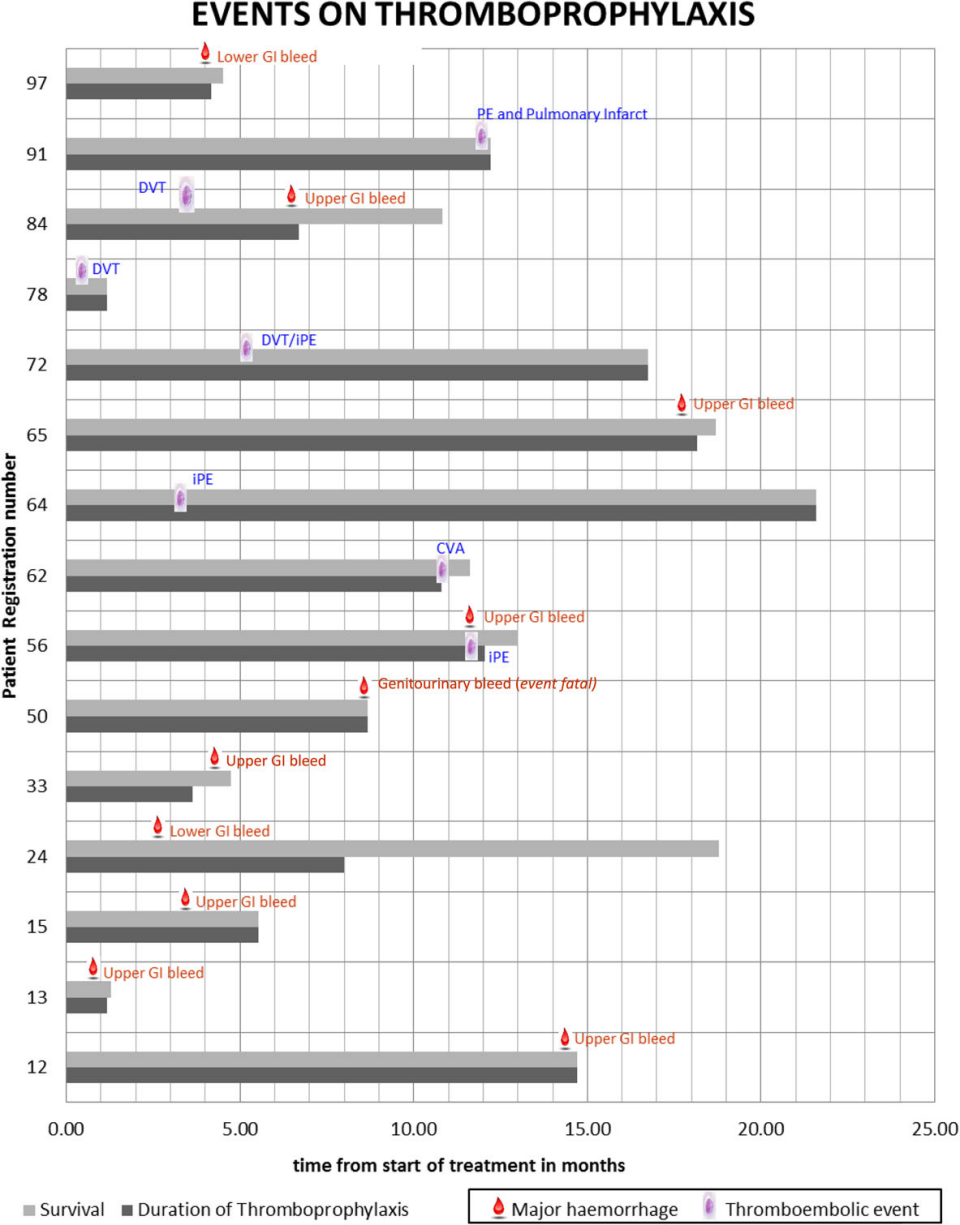
The 15 patients with events. The sequential database numbers have been retained. (GI = Gastrointestinal, iPE = Incidental Pulmonary Embolism)

Median overall survival time from commencement of IDPTP was 8.7 months, 95%CI, (6.4, 10.9). Neither thrombotic events [12.2 months, 95% CI (10.7, 13.7) Vs 8.4 months, 95% CI (6.9, 9.9) (*p* = 0.79)] nor bleeding events [median 8.7 months, 95% CI (0.4, 17) Vs 8.4 months, 95% CI (5.8, 11) (*p* = 0.94)] affected survival outcome. Progressive cancer remained the major cause of death (70 out of 82; 85%) as reported in the death certificate and agreed by the investigators. Five of these patients were found to be suffering from MB as a tumour progression-related event during the process of dying from cancer, the context often informing decision making (e.g. restraint on endoscopic or other invasive procedures or reflection on futility of transfusion). For one patient bleeding (uterine) was reported in the death certificate and confirmed by the investigators as the direct cause of death (1.2%). Two patients suffered a fatal thrombo-embolic event (2.4%). One a CVA and one a pulmonary infarct. Figure 3 charts MB and ATTE in the relevant patients, their timing and their link to the presence or absence of IDPTP over the patient life-span.

## Discussion

In this study we show that the 12 month rate of TE (8.5%) is on a par with the TE rate seen in the experimental arm of the FRAGEM study (12%) and much lower than the control arm of FRAGEM (24%). It is also much lower than in historical series (25–35%) [20]. We also show an incidence of clinically apparent VTE rate (PE and DVT) is 4.8%, similar to CONKO-04 (5.7%) and lower than in FRAGEM (8.4%) (Table 2).

The existing RCT evidence pertains to patients receiving first line chemotherapy Gemcitabine either as a single agent or in combination with a platinum [8, 9]. In this study 42% of patients received the more modern combinations of FOLFIRINOX (5-Fluorouracil, Irinotecan, Oxaliplatin) or AG (Abraxane, Gemcitabine). These chemotherapy schedules do not seem to have a diminished thrombosis risk. FOLFIRINOX has a 60% increase in risk of VTE (6.6% compared to gemcitabine alone 4.1%) [21]. In the study HALO-109-202 the rate of VTE in the investigational arm (AG-PEGPH20) was 42% and control arm (Gem Abraxane) was 25% respectively in stage 1 of the trial [22]. 40 mg of enoxaparin saw no VTE reduction in either arm. Only when an enoxaparin-IDPTP schedule was implemented did VTE rate drop to 5 and 6% respectively. A LMWH scale of dose-effectiveness in pancreatic cancer patients has recently been reported and can aid in decision making on IDPTP [7].

**Fig. 4:**
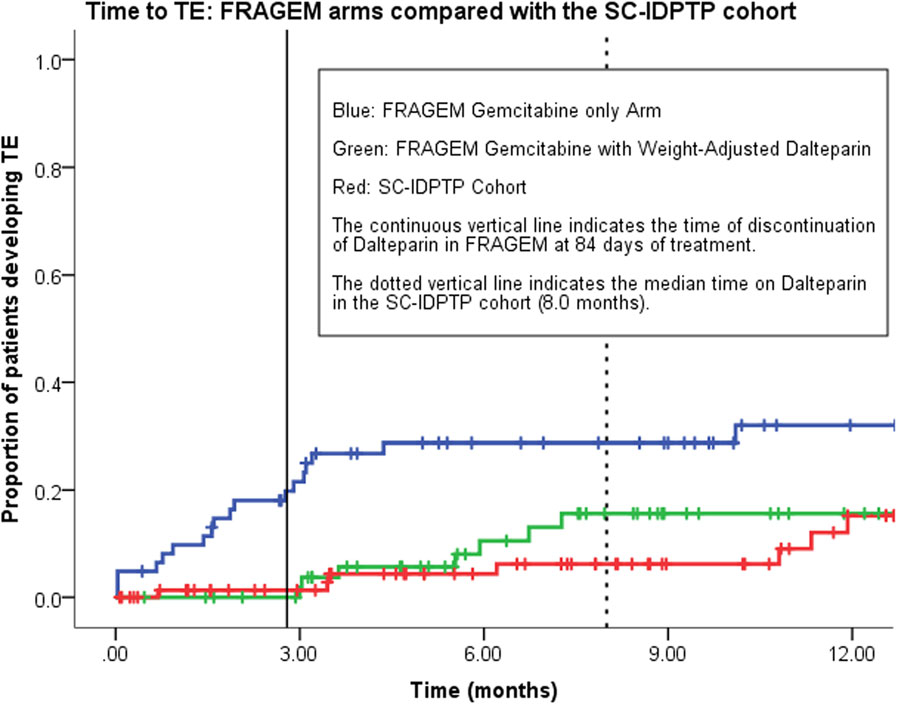
Cumulative Thromboembolic Events (TEs) graph comparing TE occurrence in the two FRAGEM study arms [8] to the TE occurrence in the SC IDPTP. (SC-IDPTP = Study Cohort of Increased Dose Primary Thromboprophylaxis, TE = Thromboembolic Event)

Of cancer patients commencing chemotherapy and who experience a VTE, 18.1% will have their first event within the first month, 47% within the first 3 months, and 72.5% within the first 6 months after starting chemotherapy [23]. In our cohort mean time to thrombosis was 6.8 months and TE-rates continue to remain diminished over the first 10 months. This is longer than seen in the FRAGEM-LMWH arm (Fig. 4). The mandatory stop of thomboprophylaxis at 84 days in FRAGEM is likely to have influenced these findings, whereas in this cohort median time on IDPTP was 8.0 months.

Figure 2a demonstrates a very late (around 12 months) manifestation of increasing TE events and may reflect the difficulties and ambivalence of maintaining thromboprophylaxis in patients arriving at the pre-terminal/terminal phase of malignancy. Adherence to anticoagulants may at that point be challenging or deemed futile. Of the 82 patients we report on, 81 had died at the time of data collection. Given the retrospective nature of the data collection for many patients we were not able to establish exact date or cause of LMWH discontinuation.

In this cohort we show that the 12 month MB rate was 12% (10/82). Sixty percent (6/10) of these related to direct (infiltration) or indirect (varices) effects of the malignancy. Of the four remaining major bleeds benign pathology was documented in 2 [uterus (fibroids) and anus (haemorrhoids)] while one upper and one lower GI bleed remained unattributed. Major bleed was an end of life feature in 5 patients but death attributable solely to bleeding was documented in only one case, while in 4 it was part of the end-of-life (EoL) process of pancreatic cancer progression being the underlying cause of death and the management of the bleed tailored to the end of life healthcare context (e.g. restraint on endoscopic or other invasive procedures). There was no survival difference between patients with either TE or bleeding compared to no events.

These data are certainly in keeping with real world data for patients with aPDAC not on thromboprophylaxis [24]. Wang et al. reported on 246 cases of aPDAC, and found an overall bleeding incidence of 13% (32 patients). A multivariate analysis showed that only stage IV disease maintained predictive significance for a bleed [HR 21.94, 95% CI (2.274–211.642)]. Haemorrhage was an end of life event in 7% (18 patients out of 246) [24] with a median time to death of 31 +/− 21 days. No impact of bleeding on survival was seen [HR 1.246,95% CI (0.754–2.060)] [8.7 months,95% CI (0.4, 17) Vs 8.4 months,95% CI (5.8, 11)].

Compliance has been shown to be better with oral agents with large early discontinuation rates for LMWHs [25]. Although injectable anticoagulants are undoubtedly more invasive than a tablet, patient views are nuanced. At least 2 studies [26, 27] have shown that patients will select anticoagulants that are least likely to interfere with their cancer treatment. In the SELECT-D sub-study [27] patients did express a preference for an oral agent, but only if this has equivalent clinical benefit to LMWH. Compliance in this study was very high, with patients staying on dalteparin for a median of 8.0 months. This may also reflect the absence of an alternative oral option in the IDPTP setting.

Very recently an experience of therapeutic dose rivaroxaban (15 mg po bd followed by 20 mg po od) in 28 aPDAC patients receiving Gemcitabine Abraxane showed a 7% major bleeding (2/28) and one breakthrough VTE (asymptomatic) 3.5% [28]. Though promising there are no randomised data in this setting for supraprophylactic DOAC schedules and one has to be aware of the greater propensity of the GI tract cancers to be associated with increased non-major but clinically relevant bleeding as seen in the recent studies of cancer associated thrombosis treatment with rivaroxaban and edoxaban [29, 30].

Two RCTs using conventional thromboprevention schedules of the direct oral anticoagulants rivaroxaban and apixaban in cancer patients have recently published [13, 14].

Patients were risk stratified using the Khorana risk stratification score. In the CASSINI study 54% of patients had either aPDAC (33%) or Gastroesophageal (GO) (21%) while in the AVERT study only 16% belonged to this group (13% aPDAC and 3% GO). Efficacy endpoint sub-analysis (prevention of VTE) for the aPDAC patients has been published only for the CASSINI study (HR 0.70, 95%CI 0.344,1.432). The latest iteration of most guidelines (2019) reinforced by the 2 recent randomised studies of DOACS, have formulated thromboprophylaxis recommendations for aPDAC [10, 11]. As the data stand LMWH-IDPTP schedules remain a proven approach for aPDAC while for the DOACs there remains the need for a well-designed single cancer RCT [31].

Our study has shortcomings; the obvious one being that this is a retrospective series and some endpoint comparisons are made to historical data. A further weakness is that we cannot account for selection bias, as we cannot identify and report in retrospect on outcomes of patients that were not offered IDPTP or not switched to IDPTP either due to contraindication or due to omission. Whilst the chemotherapy regimens were mostly gemcitabine based they included patients on different trials with experimental combinations and at least 18% on triple combination FOLFIRINOX. Nevertheless over this period, and contrary to many other cancers, the overall treatment of pancreatic cancer has brought little material change to outcomes. The study is too small to pick up substantial signals of mortality due to adverse events. Our median survivorship for the group is similar with that expected for aPDAC patients as seen in other series and studies. Although the IDPTP-dalteparin guideline adhered to in this study concurs with the CONKO-04 approach of ‘lifelong’ thromboprophylaxis, the higher doses used throughout in our study compared to CONKO-04 (40 mg enoxaparin) do not allow direct comparisons. We demonstrate VTE control but note that thromboprophylaxis at late stages of advancing pancreatic cancer where bleeding becomes a more common event should be approached cautiously and the use of IDPTP beyond the 3 months used in the major studies should be reviewed on a case by case basis.

## Conclusion

In conclusion we demonstrate that, an IDPTP-LMWH with dalteparin reflecting the evidence can be implemented and patients can adhere to it. IDPTP-LMWH was associated with lower ATTE occurrence rates than expected and comparable major bleeding rates. ATTE and MB were late events; the majority of MB was from the GI tract and the direct or indirect result of locally progressing aPDAC. Since these conditions can frequently arise in aPDAC, IDPTP-LMWH beyond 3 months needs to be approached cautiously and should be regularly reviewed due to the increasing risk of GI bleeding in late –progressing- pancreatic cancer.

## Data Availability

Database of all 101 patients and statistical analyses available upon request and authorization from HUTH R&D.

## Abbreviations

AG: Abraxane, Gemcitabine
aPDAC: Advanced Pancreatic Ductal Adenocarcinoma
ATTE: All Type Thromboembolic Events
BFI: Bleeding-free interval
DOAC: Direct Oral Anticoagulant
DVT: Deep Vein Thrombosis
ECOG-PS: Eastern Cooperative Group Performance status
FOLFIRINOX: Folinic Acid, 5-Fluorouracil, Irinotecan, Oxaliplatin
GI: Gastrointestinal
GO: Gastro-oesophageal
IPE: Incidental Pulmonary Embolism
IDPTP: Increased Dose Primary Thromboprophylaxis
LMWH: Low Molecular Weight Heparin
MB: Major Bleeding
NNT: Numbers Needed to Treat
PE: Pulmonary Embolism
PEGPH20: Pegvorhyaluronidase Alfa
TE: Thromboembolic Event
TFI: TE-free Interval
VTE: Venous Thromboembolism
